# Maternal weight trajectories and associations with infant growth in South African women

**DOI:** 10.1186/s12889-023-16963-3

**Published:** 2023-10-20

**Authors:** Hlengiwe P. Madlala, Angela M. Bengtson, Luke Hannan, Thokozile R. Malaba, Emma Kalk, Dorothy Nyemba, Andrew Boulle, Landon Myer

**Affiliations:** 1 Division of Epidemiology and Biostatistics, School of Public Health, University of Cape Town, Western Cape, Cape Town, South Africa; 2 Department of Epidemiology, School of Public Health, Brown University, Providence, RI USA; 3 Centre for Infectious Disease Epidemiology and Research, School of Public Health, University of Cape Town, Western Cape, Cape Town, South Africa; 4 Health Impact Assessment Unit, Western Cape Department of Health, Cape Town, South Africa

**Keywords:** Maternal weight, HIV infection, Pregnancy, Infant growth, Latent-class growth modelling

## Abstract

**Background:**

Despite the close relationship between pre-pregnancy body mass index (BMI), gestational weight gain (GWG) and postpartum weight (PPW), these factors are often studied separately. There are no data characterising longitudinal weight trajectories among pregnant and postpartum women in urban African populations. We examined maternal weight trajectories from pregnancy through to 12 months postpartum, factors associated with higher weight trajectory class membership and associations of weight trajectories with infant growth at 12 months.

**Methods:**

Data from 989 women were examined for weight trajectories from first antenatal care visit in pregnancy to 12 months postpartum using latent-class growth models. Baseline factors associated with class membership were assessed using multinomial logistic regression. Of the enrolled women, 613 of their infants were assessed for growth at 12 months. Anthropometry measurements for mothers and infants were conducted by a trained study nurse. Associations between maternal weight trajectory class and infant weight-for-age (WAZ), length-for-age (LAZ), weight-for-length (WLZ) at 12 months of age were analysed using linear regression.

**Results:**

Four distinct classes of maternal weight trajectories were identified. The classes included *consistent low* (29%), *consistent medium* (37%), *medium–high* (24%) and *consistent high* (10%) trajectories. Similar to trends observed with *medium–high* trajectory, baseline factors positively associated with *consistent high* class membership included age (OR 1.05, 95% CI 1.01–1.09), pre-pregnancy BMI (OR 2.24, 95% CI 1.97–2.56), stage 1 hypertension (OR 3.28, 95% CI 1.68–6.41), haemoglobin levels (OR 1.39, 95% CI 1.11–1.74) and parity (OR 1.39, 95% CI 1.15–1.67); living with HIV (OR 0.47, 95% CI 0.30–0.74) was inversely associated. In adjusted analyses, compared to *consistent medium* weight trajectory, *consistent low* weight trajectory (mean difference -0.41, 95% CI -0.71;-0.12) was associated with decreased, and *consistent high* weight trajectory (mean difference 1.21, 95% CI 0.59–1.83) with increased infant WAZ at 12 months of age.

**Conclusion:**

Identification of unique longitudinal weight trajectory groupings might inform comprehensive efforts targeted at improving healthy maternal weight and infant outcomes.

**Supplementary Information:**

The online version contains supplementary material available at 10.1186/s12889-023-16963-3.

## Introduction

In recent years, low-middle-income countries are increasingly facing a dual burden of malnutrition characterised by both undernutrition and overnutrition [1]. This trend is particularly prominent among women in rural settings, with some continuing to experience underweight while others are overweight and obese [2]. Undernutrition is partly attributed to persistent poverty and household food insecurity while overnutrition is partly attributed to increased consumption of inexpensive high calorie dense foods accompanied by a sedentary lifestyle [3, 4]. In sub-Saharan Africa (SSA), undernutrition is mainly observed among people living with HIV due to HIV infection itself and the risk of opportunistic infections [5]. However, obesity is also highly common in women living with HIV (WLHIV) due to exposure to the ‘obesogenic’ environment faced by the general public [6, 7]. In addition, some classes of antiretroviral therapy (ART) are implicated in contributing to weight gain among the HIV group [8, 9]. Therefore, we compared weight trajectories in WLHIV and without HIV.

Weight status in women of reproductive age influences maternal and infant health outcomes. Elevated pre-pregnancy weight and excessive gestational weight gain (GWG) increase the risk of gestational diabetes and hypertension, including pre-eclampsia and eclampsia, as well as complicated delivery and caesarean Sects [10–13]. Further, pre-pregnancy obesity and excessive GWG is linked to postpartum weight (PPW) retention, which is recognised as an important predictor of non-communicable diseases (NCDs) in mid-life including cardiovascular diseases (CVDs) [14–17]. Pregnant women with elevated weight are also at risk of having stillbirths, preterm as well as large for gestational age (LGA) infants [18, 19]. On the other hand, underweight weight in pregnancy is linked to anaemia which is highly prevalent in South Africa and associated with hypertensive disorders [20, 21]. In addition, underweight increases the risk of having preterm and small size for gestational age (SGA) infants [22, 23]. Although some preterm and SGA infants remain small throughout their childhood [24, 25], others experience rapid ‘catch-up’ growth; both scenarios are associated with childhood obesity and increased lifetime risk of CVDs [26–29]. Likewise, most data report that LGA infants experience obesity and related-metabolic complications later in life [30, 31], while some report a compensatory slower growth trajectory [32, 33].

Despite the evident contribution of not only pre-pregnancy BMI but also GWG and PPW to poor maternal health outcomes, there are no studies in SSA that have applied a trajectory approach to understand weight changes over the perinatal period and implications for infant growth. Therefore, the objective of this study was to examine maternal weight trajectories from pregnancy through to 12 months postpartum, factors associated with higher weight trajectory class membership and associations of weight trajectories with infant growth at 12 months.

## Methods

### Study design and population

For this prospective cohort study, consecutive pregnant women attending antenatal care (ANC) services at the Gugulethu Community Health Centre in Cape Town, South Africa were enrolled between January 2017 and July 2018. Eligible women were those who were ≥ 18 years of age and presenting for the first time for ANC regardless of gestational age. Women living with HIV had either initiated efavirenz-based ART before conception or intended to initiate ART during pregnancy following their first ANC visit. Gugulethu is a peri-urban community with a population of ~ 300 000, with predominantly low socioeconomic status (SES) and HIV prevalence of 30% among pregnant women [34–36]. Pregnant women were prospectively followed through face-to-face interviews with intensive anthropometry measurements that took place during the first, second and third visit conducted in pregnancy; and were followed-up with their infants at < 7 days, 10 weeks, 6 months and 12 months postpartum. All 989 women enrolled in the cohort were included for the weight trajectory analysis, however 613 of 907 (68%) live singleton infants were included for infant growth analysis. Excluded infants (*n* = 376) were twins (*n* = 14), miscarriage/stillbirths/terminations (*n* = 58), neonatal/infant/maternal death (*n* = 10), maternal/infant seroconversion to HIV (*n* = 11), no longer interested (*n* = 36), relocated (*n* = 50), lost to follow-up (*n* = 129) and mom attended 12 months visit without baby (*n* = 68) (Figure S1).

### Outcome assessment

The primary outcome of interest was maternal weight trajectory from first ANC visit through 12 months postpartum. Weight measurements from enrolment in pregnancy through to 12 months postpartum were conducted by a trained study nurse using a calibrated scale (Charder, Taichung City, Taiwan) accurate to within 0.5 kg. A study nutritionist repeated anthropometric training for the study nurse at regular intervals, with structured and supervised competency. A total of up to 7 weight assessments (first, second and third visits in pregnancy; and at < 7 days, 10 weeks, 6 and 12 months postpartum periods) were used to examine weight trajectories. Missing weights at the first visit were 0.01%, second visit were 36% and third visit were 51% in pregnancy; and at < 7 days were *n* = 42%, 10 weeks were 38%, 6 months were 35% and 12 months were 30% postpartum. Participants with missing datapoints were included in the model, the model created a trajectory using timepoints with available data and excluding timepoints with missing data [37, 38]. The secondary outcome of interest was infant anthropometry Z-scores (WAZ, LAZ and WLZ) at 12 months calculated using WHO child growth standards [39]. Child anthropometry assessments (weight and length) at 12 months of age were performed by the same trained study nurse who conducted maternal anthropometry measurements following a standardised protocol based on WHO guidelines [39].

### Other characteristics

At enrolment, height measurements were taken to the nearest 0.1 cm using a stadiometer (Seca, Birmingham, United Kingdom). Using standard GWG charts developed using estimated gestational age (GA) at measurement and related BMI categories, we applied a correction factor on weight measured at first ANC visit to estimate pre-pregnancy BMI using a method described by Santos et al. [40]. Briefly, using international standards for GWG in pregnancy [40], the median weight gained for each week of gestation was subtracted from the weight measured at first ANC visit based on BMI category as GWG differs according to pre-pregnancy BMI. Estimated pre-pregnancy BMI was then calculated as GA-corrected weight divided by squared height; and was categorised as underweight (< 18.5), normal (18.5–24.9), overweight (25–29.9) and obese (≥ 30) in kg/m^2^. Weekly maternal GWG was calculated by dividing the weight change between enrolment and second or third visits by the number of weeks lapsed between the two intervals [41], and expressed as kg/week. Rate of GWG was categorised as slow, normal and fast based on the Institute of Medicine (IOM) recommended median GWG (IQR) which vary by BMI category [42]. Postpartum weight change was calculated as the difference between measured 12 months postpartum weight and estimated pre-pregnancy weight, and was categorised as loss (< 0 kg), stable (0–4.9kg) and gain (≥ 5 kg) [43].

Maternal socio-demographic and clinical data were collected via interviewer-administered questionnaires. SES was a composite score based on level of education, employment status, type of housing, and presence of a toilet, running water, electricity, fridge, telephone, and television in the house [44]; participants were categorised into tertiles corresponding to lowest, middle and highest SES group. Baseline blood pressure (BP) and haemoglobin (Hb) measurements were performed by healthcare providers as part of routine care at the first ANC visit. BP was categorised as: normal (< 120/80 mmHg), elevated (systolic 120–129 and diastolic < 80 mmHg), stage 1 hypertension (systolic 130–139 or diastolic 80–89 mmHg) or stage 2 hypertension (systolic ≥ 140 or diastolic ≥ 90 mmHg) [45]. Hb was categorised as normal (≥ 11.0), mild anaemia (10–10.9), moderate anaemia (7–9.9) or severe anaemia (< 7) in g/dL [46]. Alcohol use was assessed using AUDIT questionnaire and was defined as ‘yes’ if indicated alcohol use since finding out about the current pregnancy. Breastfeeding duration was obtained through self-report and categorised as ‘never’, ‘ever’; those who ‘ever’ breastfed were further categorised into < 6 months and ≥ 6 months duration of any intensity. Food intake was assessed at first ANC via a 7-day recall food frequency questionnaire with responses categorised as ‘never’, ‘1–3’ and ‘4–7’ days. Parity, HIV status (with HIV and without HIV), ART initiation timing (before pregnancy and during pregnancy) and CD4 count were abstracted from medical records. Relationship status was self-reported and categorised as ‘not in a relationship’, ‘not cohabiting/married but not living together’ and ‘cohabiting/married and living together’.

Gestational age (GA) was determined by ultrasound operated by an experienced research sonographer at the first study visit. Delivery GA was calculated by adding the number of weeks elapsed between enrolment and delivery dates to the GA measured at first ANC visit. Delivery GA was categorised as term (≥ 37 weeks) or preterm delivery (PTD, < 37 weeks) [47]. Preterm delivery was further categorised into spontaneous and medically-indicated PTD based on delivery mode information abstracted from medical records. Spontaneous PTD was defined as onset of labour and spontaneous vaginal delivery at less than 37 weeks GA, while medically-indicated PTD was defined as delivery at less than 37 weeks after induction of labour or by caesarean section. Neonatal data at delivery including birth weight and gender were obtained from medical records. Infant birth weight was categorised as low (< 2500), normal (2500 – 4000) and high (> 4000) birthweight in grams [48]. Size for GA was calculated based on infant GA, birthweight and sex using INTERGROWTH-21^st^ standards and categorised as small (< 10^th^), appropriate (10 – 90^th^) and large (> 90^th^) for GA in percentiles [49].

### Statistical analysis

Latent-class growth modelling was used to create maternal weight trajectory classes from pre-pregnancy through to 12 months postpartum period. This method allows longitudinal data to inform groupings of individuals following distinct patterns over time, enabling those who are LTFU to also contribute available data [37, 38]. Latent-class growth models were fit with random effects for each participant and fixed effects for time period. A minimum of three classes was considered a priori. However, model fit based on Bayesian Information Criterion (BIC) values was used to guide the final number of classes included. We evaluated baseline factors associated with maternal weight trajectory class using univariate logistic regression. These baseline factors are typically collected at the first ANC visit in this setting. The factors included maternal age (continuous and categorical), estimated pre-pregnancy BMI (continuous), BP (continuous and categorical), Hb (continuous and categorical), education (categorical), SES (categorical), relationship status (categorical), alcohol use (categorical), GA at first ANC (continuous and categorical), parity (continuous and categorical), HIV status (categorical), ART initiation timing (categorical) and past 7-day food frequency (categorical). Because pre-pregnancy BMI is a known predictor of weight progression in pregnancy and postpartum, we performed BMI-adjusted analyses to assess weight trajectory predictors that are independent of BMI. To examine the association between maternal weight trajectory class and infant WAZ, LAZ and WLZ at 12 months, univariable and multivariable linear regression were used. Multivariable models for infant growth were adjusted for maternal (estimated pre-pregnancy BMI, parity, SES and alcohol use) and infant (sex, delivery GA and feeding duration) factors based on the basis of existing literature, theoretical and conceptual reasoning. Having observed high obesity in WLHIV in our previous studies [50, 51], we tested the hypothesis that weight trajectories in WLHIV will not differ from those of women without HIV, hence we stratified the analysis by HIV status. In all analyses, a *p*-value < 0.05 was considered statistically significant. With the exception of maternal weight trajectory class analyses which were created using R studio, all other analyses were performed using STATA version 15.0 (Stata Corporation, College Station, TX, USA).

## Results

A total of 989 women (48% WLHIV) were included in the analyses for maternal weight trajectories. At first ANC entry, the median GA was 20 weeks (IQR, 14–25), maternal median age was 29 years (IQR, 25–34) and 25% were primiparous. Median BMI was 29 kg/m^2^ (IQR, 24–34): 26 (2%) underweight, 273 (28%) normal, 244 (25%) overweight and 428 (43%) obese BMI. Median GWG rate was 0.36 kg/week (IQR, 0.14–0.54): 264 (27%) slow, 121 (12%) normal, 361 (37%) fast GWG rate. Median PPW at 12 months was 3.87 kg (IQR, -1.10–9.04): 209 (21%) weight loss, 178 (18%) stable weight, 300 (30%) high weight retention (Table 1).

**Table 1:** Characteristics of women included in the analysis, overall and stratified by maternal HIV status

		HIV status		
	Overall *N* = 989 (100%) N (%)	Without HIV *N* = 510 (52%) N (%)	With HIV *N* = 479 (48%) N (%)	*p*-value
Age (years)				** < 0.01**
< 24	240 (24)	171 (34)	69 (14)	
25–29	292 (30)	157 (31)	135 (28)	
30–34	254 (26)	109 (21)	145 (30)	
≥ 35	203 (21)	73 (14)	130 (27)	
Median (IQR)	29 (25–34)	27 (23–32)	31 (26–35)	
Blood pressure (mmHg)				0.32
Normal	620 (63)	309 (61)	311 (65)	
Elevated	160 (16)	93 (18)	67 (14)	
Stage 1 hypertension	108 (11)	56 (11)	52 (11)	
Stage 2 hypertension	101 (10)	52 (10)	49 (10)	
Median SBP (IQR)	113 (105–122)	114 (105–122)	113 (105–122)	
Median DBP (IQR)	67 (61–73)	66 (60–73)	67 (61–73)	
Haemoglobin (g/dL)				0.21
Normal (≥ 11.0)	394 (40)	215 (42)	179 (37)	
Mild anaemia (10–10.9)	150 (15)	74 (15)	76 (16)	
Moderate anaemia (7–9.9)	114 (12)	48 (9)	66 (14)	
Severe anaemia (< 7)	6 (1)	3 (1)	3 (1)	
Missing	325 (33)	170 (33)	155 (32)	
Median (IQR)	11.3 (10.4–12.1)	11.4 (10.5–12.1)	11.2 (10.2–12.0)	
Education				**0.02**
Primary	37 (4)	15 (3)	22 (5)	
High school	929 (94)	477 (94)	452 (94)	
Tertiary	23 (2)	18 (4)	5 (1)	
Socio-economic status				0.15
Lower	325 (33)	152 (30)	173 (36)	
Middle	274 (28)	142 (28)	132 (28)	
Higher	388 (39)	215 (42)	173 (36)	
Missing	2 (0.2)	1 (0.2)	1 (0.2)	
Relationship status				0.78
No relationship	46 (5)	23 (5)	23 (5)	
Not Cohabiting/married-NLT	509 (51)	267 (52)	242 (51)	
Cohabiting/married-LT	428 (43)	218 (43)	210 (44)	
Missing	6 (1)	2 (0.4)	4 (1)	
*Alcohol use				0.99
No	898 (91)	463 (91)	435 (91)	
Yes	89 (9)	46 (9)	43 (9)	
Missing	2 (0.2)	1 (0.2)	1 (0.2)	
GA at first ANC (weeks)				0.22
1^st^ trimester (≤ 13)	229 (23)	109 (21)	120 (25)	
2^nd^ trimester (14–28)	609 (62)	314 (62)	295 (62)	
3^rd^ trimester (> 28)	123 (12)	73 (14)	50 (10)	
Missing	28 (3)	14 (3)	14 (3)	
Median (IQR)	20 (14–25)	21 (15–26)	19 (13–24)	
Parity				** < 0.01**
Primiparity	251 (25)	168 (33)	83 (17)	
Multiparity	738 (75)	342 (67)	396 (83)	
Median (IQR)	1 (0–2)	1 (0–2)	1 (1–2)	
ART initiation timing				
Pre-pregnancy	291 (61)	––-	291 (61)	
During pregnancy	188 (39)	––-	188 (39)	
CD4 count (cells/µL)				
Median (IQR)	458 (311–604)	––-	458 (311–604)	
Missing	56 (12)	––-	56 (12)	
Estimated pre-pregnancy BMI (kg/m^2^)				**0.01**
Underweight (< 18.5)	21 (2)	16 (3)	5 (1)	
Normal (18.5–24.9)	273 (28)	136 (27)	137 (29)	
Overweight (25–29.9)	244 (25)	110 (22)	134 (28)	
Obese (≥ 30)	428 (43)	237 (46)	191 (40)	
Missing	23 (2)	11 (2)	12 (3)	
Median (IQR)	29 (24–34)	29 (24–37)	28 (24–33)	
Gestational weight gain rate (kg/week)				**0.01**
Slow	264 (27)	110 (22)	154 (32)	
Normal	121 (12)	54 (11)	67 (14)	
Fast	361 (37)	199 (39)	162 (34)	
Missing	243 (25)	147 (29)	96 (20)	
Median (IQR)	0.36 (0.14–0.54)	0.40 (0.17–0.57)	0.31 (0.10–0.50)	
Postpartum weight change at 12 months (kg)				**0.02**
Weight loss (< 0 kg)	209 (21)	95 (19)	114 (24)	
Stable weight retention (0–4.9 kg)	178 (18)	81 (16)	97 (20)	
High weight retention (≥ 5 kg)	300 (30)	169 (33)	131 (27)	
Missing	302 (31)	165 (32)	137 (29)	
Median (IQR)	3.87 (-1.10–9.04)	4.69 (-0.55–9.99)	3.03 (-2.05–7.75)	
Food intake in past 4–7 days				0.91
Starch	523 (53)	266 (53)	275 (54)	
Protein	285 (29)	167 (33)	118 (25)	
Dairy	177 (18)	88 (17)	89 (19)	
Fruits	131 (13)	63 (12)	68 (14)	
Vegetables	289 (29)	150 (29)	139 (29)	
Legumes	79 (8)	38 (7)	41 (9)	
Oils	386 (39)	198 (39)	188 (39)	
Missing	3 (0.3)	2 (0.4)	1 (0.2)	

### Maternal weight trajectories

**Fig. 1:**
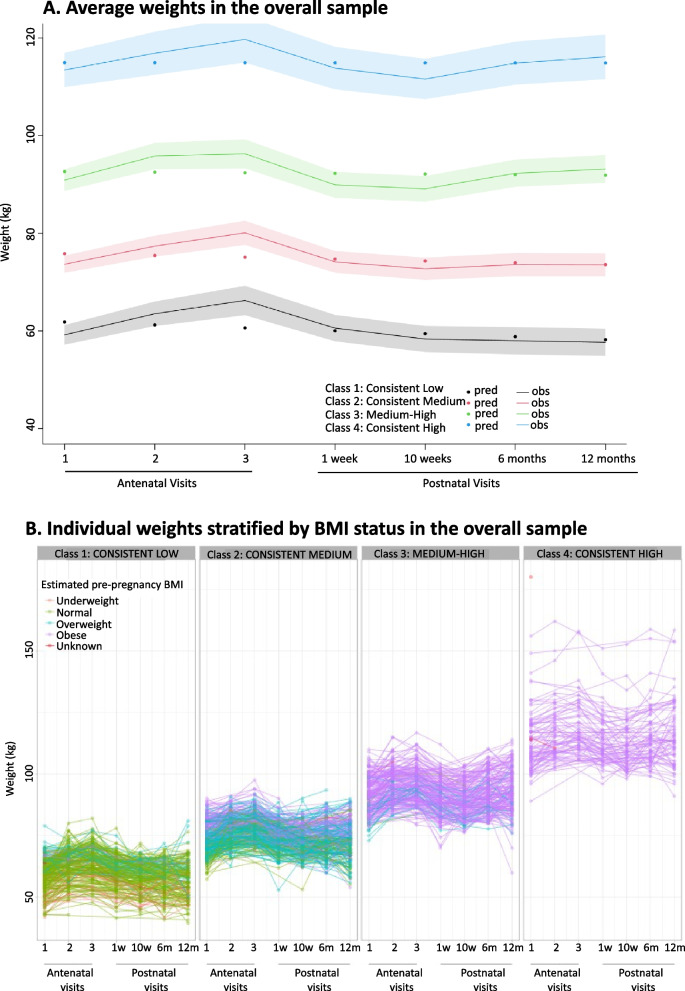
Four distinct maternal weight trajectory classes in the overall sample, A. average weights and B. individual weights by estimated pre-pregnancy BMI. Notably, normal BMI women in *consistent low* class had significantly lower mean GWG (0.41 vs 0.58 kg/week, *p* = 0.01) and PPW change (3.54 vs 8.94 kg, *p* = 0.01) compared to normal BMI women in *consistent medium* class (Fig. 2). Overweight BMI women in *consistent medium* class had significantly lower mean GWG (0.37 vs 0.84 kg/week, *p* = 0.01) and PPW (3.94 vs 13.58 kg, *p* = 0.01) compared to overweight BMI women in *medium–high* class. Although non-significant, obese BMI women in *medium–high* class had lower mean GWG (0.31 vs 0.42 kg/week, *p* = 0.46) compared to obese BMI women in *consistent high* class

Four distinct maternal weight trajectory classes were identified and included in the analysis. This decision was based on BIC values which did not change substantially beyond the 4^th^ class. To assign individuals into a particular class, the model used the class with the highest predicted probability out of the 4 classes for that individual [37, 38]. The probability cut-off for assigning a class to an individual was high at 0.90. The trajectories were named based on initial weight in pregnancy and the pattern observed at the postpartum period; class 1 (*consistent low*), class 2 (*consistent medium*), class 3 (*medium–high*) and class 4 (*consistent high*). The 4 trajectories started off at different mean weights in the first visit (*consistent low* [59kg], *consistent medium* [74kg], *medium–high* [91kg], *consistent high* [114kg], *p*-value 0.01). However, all trajectories displayed the same pattern of steady increase in weight in second and third visits, followed by a decline at < 7 days after delivery as expected (Fig. 1A). From 10 weeks through 12 months postpartum, the weight remained stable for *consistent low* and *consistent medium*; but it increased for *medium–high* and *consistent high*.

**Fig. 2:**
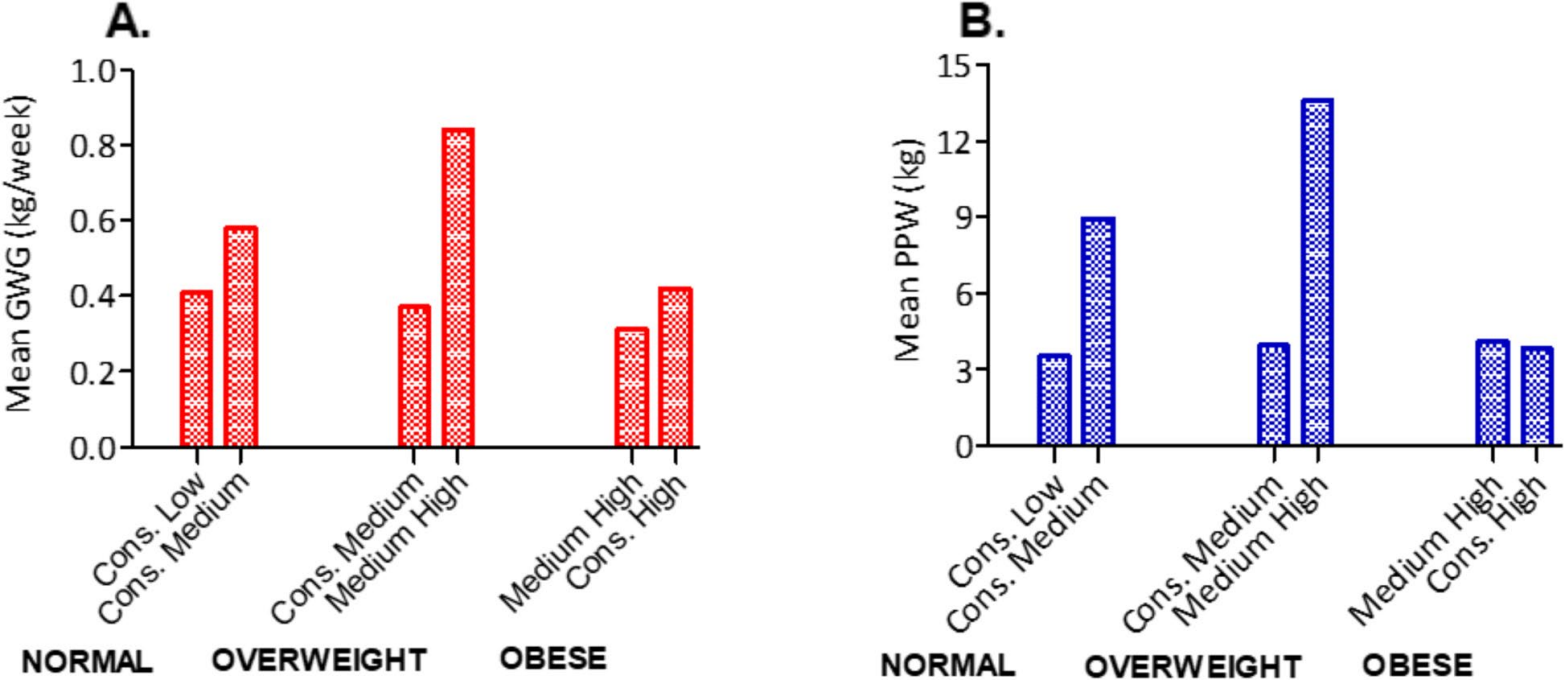
Illustration of the differences in mean GWG (A) and PPW (B) among women who had similar estimated pre-pregnancy BMI categories but different weight trajectories. The proportion of women with normal/overweight/obese BMI is indicated on top of each trajectory class bar

*Consistent low* (29%) was composed of women who had underweight (7%), normal (72%), overweight (15%), obese (2%) and unknown (4%) estimated pre-pregnancy BMI at baseline, the majority of these women gained weight at either low/normal rate (per GWG [kg/week] IOM guidelines) during pregnancy and either lost/retained a stable (< 0 to 4.9kg) amount of weight postpartum (Fig. 1B). *Consistent medium* (37%) was composed of women who had normal (19%), overweight (49%), obese (30%) and unknown (2%) estimated pre-pregnancy BMI, the majority of these women gained weight at a low/normal rate (per GWG [kg/week] IOM guidelines) during pregnancy and either lost/retained a stable (< 0 to 4.9kg) amount of weight postpartum. *Medium–high* (24%) was composed of women who had normal (0.5%), overweight (9%), obese (90%) and unknown (0.5%) estimated pre-pregnancy BMI, the majority of these women gained weight at a normal/elevated rate (per GWG [kg/week] IOM guidelines) during pregnancy and retained either a stable/excess (0 to > 5.00 kg) amount of weight postpartum. *Consistent high* (10%) was composed of women who had obese (97%) and unknown (3%) estimated pre-pregnancy BMI, the majority of these women gained weight at a faster rate (per GWG [kg/week] IOM guidelines) during pregnancy and retained an excess (≥ 5.00 kg) amount of weight postpartum. The weight trajectory pattern for all 4 membership classes was similar in both women living with and without HIV (Figure S2).

### Predictors of maternal weight trajectory class

In anadjusted analysis (Table 2), continuous baseline factors positively associated with *consistent high* class membership included age (OR 1.05, 95% CI 1.01–1.09), estimated pre-pregnancy BMI (OR 2.24, 95% CI 1.97–2.56), systolic BP (OR 1.04, 95% CI 1.02–1.06), diastolic BP (OR 1.05, 95% CI 1.05–1.08), Hb levels (OR 1.39, 95% CI 1.11–1.74) and parity (OR 1.39, 95% CI 1.15–1.67). Categorical baseline factors associated with *consistent high* class membership included BP, Hb, parity and living with HIV. In particular, compared to normal BP, elevated BP (OR 2.06, 95% CI 1.16–3.67), stage 1 hypertension (OR 3.28, 95% CI 1.68–6.41) and stage 2 hypertension (OR 2.12, 95% CI 1.09–4.10) were positively associated; compared to normal Hb, moderate anaemia (OR 0.44, 95% CI 0.19–0.99) was negatively associated; compared to primiparity, multiparity (OR 2.30, 95% CI 1.20–4.41) was positively associated; compared to not living with HIV, living with HIV (OR 0.47, 95% CI 0.30–0.74) was negatively associated.

**Table 2:** Logistic regression for the association between baseline maternal characteristics and weight trajectory class (reference = *consistent medium*), unadjusted and adjusted for estimated pre-pregnancy BMI

		***CONSISTENT LOW***		***MEDIUM–HIGH***		***CONSISTENT HIGH***	
	Overall *N* = 989 (100%) N (%)	Unadjusted OR (95% CI)	Estimated pre-pregnancy BMI Adjusted aOR (95% CI)	Unadjusted OR (95% CI)	Estimated pre-pregnancy BMI Adjusted aOR (95% CI)	Unadjusted OR (95% CI)	Estimated pre-pregnancy BMI Adjusted aOR (95% CI)
Age (years)							
< 24	240 (24)	1.00 (Ref)	1.00 (Ref)	1.00 (Ref)	1.00 (Ref)	1.00 (Ref)	1.00 (Ref)
25–29	292 (30)	0.88 (0.59–1.32)	0.84 (0.49–1.47)	**1.97 (1.10–3.54)**	**3.87 (1.61–9.30)**	0.86 (0.27–2.71)	1.83 (0.48–6.94)
30–34	254 (26)	0.82 (0.54–1.26)	1.04 (0.62–1.77)	1.38 (0.75–2.57)	1.64 (0.72–3.76)	1.06 (0.35–3.21)	1.45 (0.42–5.01)
≥ 35	203 (21)	**0.33 (0.20–0.55)**	0.64 (0.34–1.23)	1.16 (0.65–2.08)	2.51 (0.98–6.40)	0.91 (0.30–2.80)	1.75 (0.45–6.86)
Median (IQR)	29 (25–34)	**0.94 (0.91–0.96)**	0.98 (0.95–1.02)	1.01 (0.99–1.04)	0.99 (0.96–1.03)	**1.05 (1.01–1.09)**	1.00 (0.94–1.06)
Estimated pre-pregnancy BMI (kg/m^2^)							
Median (IQR)	29 (24–34)	**0.61 (0.56–0.65)**	1.00 (Ref)	**1.55 (1.42–1.69)**	1.00 (Ref)	**2.24 (1.97–2.56)**	1.00 (Ref)
Blood pressure (mmHg)							
Normal	620 (63)	1.00 (Ref)	1.00 (Ref)	1.00 (Ref)	1.00 (Ref)	1.00 (Ref)	1.00 (Ref)
Elevated	160 (16)	**0.52 (0.32–0.85)**	0.86 (0.46–1.59)	**1.61 (1.04–2.48)**	1.58 (0.83–3.00)	**2.06 (1.16–3.67)**	1.53 (0.54–4.37)
Stage 1 hypertension	108 (11)	0.79 (0.43–1.43)	1.24 (0.57–2.66)	**2.63 (1.56–4.43)**	**2.21 (1.17–4.17)**	**3.28 (1.68–6.41)**	2.20 (0.76–6.38)
Stage 2 hypertension	101 (10)	0.77 (0.46–1.30)	1.49 (0.68–3.27)	0.84 (0.46–1.52)	0.67 (0.33–1.33)	**2.12 (1.09–4.10)**	1.11 (0.35–3.47)
Median SBP (IQR)	113 (105–122)	**0.97 (0.95–0.98)**	0.98 (0.96–1.00)	**1.02 (1.01–1.03)**	1.01 (1.00–1.03)	**1.04 (1.02–1.06)**	1.03 (1.00–1.05)
Median DBP (IQR)	67 (61–73)	**0.98 (0.63–0.99)**	1.00 (0.97–1.02)	**1.03 (1.01–1.05)**	1.02 (0.99–1.04)	**1.05 (1.02–1.08)**	1.03 (0.99–1.08)
Haemoglobin (g/dL)							
Normal (≥ 11.0)	394 (40)	1.00 (Ref)	1.00 (Ref)	1.00 (Ref)	1.00 (Ref)	1.00 (Ref)	1.00 (Ref)
Mild anaemia (10–10.9)	150 (15)	1.28 (0.80–2.05)	0.93 (0.51–1.67)	0.72 (0.44–1.18)	0.77 (0.38–1.56)	0.50 (0.24–1.02)	0.44 (0.14–1.36)
Moderate anaemia (7–9.9)	114 (12)	1.38 (0.84–2.27)	0.80 (0.43–1.50)	**0.42 (0.23–0.78)**	0.78 (0.37–1.65)	**0.44 (0.19–0.99)**	0.44 (0.12–1.54)
Severe anaemia (< 7)	6 (1)	6.46 (0.71–58.87)	**12.21 (3.03–49.18)**	1.20 (0.07–19.40)	**0.08 (0.01–0.54)**	0.01 (1.78^–6^-0.01)	**1.14**^**–7**^** (2.05**^**–8**^**-6.32**^**–7**^**)**
Median (IQR)	(10.4–12.1)	**0.86 (0.76–0.98)**	1.02 (0.87–1.21)	**1.15 (0.99–1.34)**	1.15 (0.94–1.40)	**1.39 (1.11–1.74)**	**1.58 (1.10–2.25)**
Education							
Primary	37 (4)	1.00 (Ref)	1.00 (Ref)	1.00 (Ref)	1.00 (Ref)	1.00 (Ref)	1.00 (Ref)
High school	929 (94)	1.38 (0.60–3.18)	1.42 (0.46–4.35)	1.75 (0.67–4.54)	2.25 (0.95–5.33)	0.73 (0.28–1.92)	0.84 (0.27–2.62)
Tertiary	23 (2)	1.78 (0.47–6.72)	1.18 (0.21–6.55)	2.67 (0.65–10.89)	2.34 (0.42–13.00)	0.76 (0.12–4.76)	0.47 (0.02–12.80)
Socio-economic status							
Lower	325 (33)	1.00 (Ref)	1.00 (Ref)	1.00 (Ref)	1.00 (Ref)	1.00 (Ref)	1.00 (Ref)
Middle	274 (28)	0.89 (0.60–1.07)	1.13 (0.66–1.95)	1.19 (0.78–1.83)	1.49 (0.84–2.64)	0.76 (0.43–1.34)	1.30 (0.48–3.46)
Higher	388 (39)	1.07 (0.74–1.55)	1.04 (0.63–1.73)	**1.61 (1.09–2.39)**	**2.11 (1.24–3.58)**	0.98 (0.59–1.63)	1.65 (0.69–3.94)
Relationship status							
Not Cohabiting/married-NLT	509 (51)	1.00 (Ref)	1.00 (Ref)	1.00 (Ref)	1.00 (Ref)	1.00 (Ref)	1.00 (Ref)
No relationship	46 (5)	1.21 (0.60–2.44)	1.63 (0.65–4.08)	1.30 (0.57–2.95)	0.58 (0.19–1.73)	2.59 (0.59–11.50)	0.43 (0.03–7.47)
Cohabiting/married-LT	428 (43)	0.81 (0.40–1.65)	0.87 (0.56–1.37)	1.65 (0.73–3.76)	1.13 (0.73–1.76)	3.25 (0.73–14.39)	1.42 (0.67–3.00)
^a^Alcohol use							
No	898 (91)	1.00 (Ref)	1.00 (Ref)	1.00 (Ref)	1.00 (Ref)	1.00 (Ref)	1.00 (Ref)
Yes	89 (9)	1.34 (0.80–2.24)	1.42 (0.73–2.78)	0.86 (0.47–1.56)	0.94 (0.40–2.22)	0.77 (0.33–1.80)	0.54 (0.10–2.94)
GA at first ANC (weeks)							
1^st^ trimester (≤ 13)	229 (23)	1.00 (Ref)	1.00 (Ref)	1.00 (Ref)	1.00 (Ref)	1.00 (Ref)	1.00 (Ref)
2^nd^ trimester (14–28)	609 (62)	0.86 (0.59–1.27)	0.66 (0.39–1.10)	0.77 (0.52–1.14)	0.73 (0.45–1.20)	0.51 (0.30–0.85)	0.44 (0.19–1.02)
3^rd^ trimester (> 28)	123 (12)	0.78 (0.44–1.37)	**0.34 (0.17–0.68)**	0.72 (0.40–1.30)	1.28 (0.61–2.70)	0.99 (0.50–1.95)	1.88 (0.60–5.86)
Median (IQR)	20 (14–25)	0.99 (0.97–1.01)	**0.95 (0.92–0.98)**	0.99 (0.97–1.01)	1.01 (0.98–1.04)	1.00 (0.97–1.03)	1.04 (0.99–1.09)
Parity							
Primiparity	251 (25)	1.00 (Ref)	1.00 (Ref)	1.00 (Ref)	1.00 (Ref)	1.00 (Ref)	1.00 (Ref)
Multiparity	738 (75)	**0.54 (0.38–0.76)**	0.84 (0.54–1.30)	1.15 (0.78–1.71)	1.04 (0.64–1.69)	**2.30 (1.20–4.41)**	1.47 (0.52–4.15)
Median (IQR)	1 (0–2)	**0.78 (0.67–0.92)**	0.85 (0.71–1.02)	**1.06 (0.92–1.23)**	0.99 (0.81–1.20)	**1.39 (1.15–1.67)**	1.11 (0.76–1.61)
HIV status							
Without HIV	510 (52)	1.00 (Ref)	1.00 (Ref)	1.00 (Ref)	1.00 (Ref)	1.00 (Ref)	1.00 (Ref)
With HIV	479 (48)	0.76 (0.55–1.03)	0.97 (0.63–1.48)	**0.68 (0.49–0.94)**	0.72 (0.47–1.11)	**0.47 (0.30–0.74)**	**0.45 (0.21–0.95)**
ART initiation timing							
Pre-pregnancy	291 (61)	1.00 (Ref)	1.00 (Ref)	1.00 (Ref)	1.00 (Ref)	1.00 (Ref)	1.00 (Ref)
During pregnancy	188 (39)	1.21 (0.78–2.07)	1.61 (0.88–2.92)	0.71 (0.44–1.17)	**0.49 (0.26–0.95)**	0.62 (0.29–1.33)	0.34 (0.10–1.23)
Food intake in past 4–7 days							
Starch	523 (53)	0.56 (0.35–0.91)	0.53 (0.28–1.01)	1.36 (0.75–2.49)	1.31 (0.58–2.94)	0.73 (0.36–1.48)	0.56 (0.16–1.96)
Protein	285 (29)	1.26 (0.85–1.86)	1.22 (0.73–2.03)	1.08 (0.71–1.62)	0.97 (0.56–1.68)	1.10 (0.63–1.94)	1.01 (0.39–2.62)
Dairy	177 (18)	0.70 (0.46–1.06)	0.72 (0.41–1.27)	0.80 (0.51–1.26)	0.79 (0.45–1.40)	1.14 (0.66–1.99)	0.91 (0.36–2.30)
Fruits	131 (13)	0.77 (0.48–1.22)	0.78 (0.43–1.44)	0.81 (0.50–1.33)	0.94 (0.51–1.74)	0.73 (0.37–1.44)	0.47 (0.11–1.98)
Vegetables	289 (29)	0.68 (0.46–0.99)	0.89 (0.52–1.53)	0.83 (0.56–1.23)	0.64 (0.37–1.08)	1.30 (0.77–2.21)	0.92 (0.38–2.25)
Legumes	79 (8)	0.71 (0.40–1.27)	0.61 (0.30–1.25)	0.94 (0.38–1.35)	1.12 (0.49–2.56)	0.96 (0.44–2.12)	3.02 (0.76–11.95)
Oils	386 (39)	0.78 (0.54–1.13)	0.86 (0.52–1.41)	0.91 (0.62–1.34)	0.74 (0.46–1.21)	0.92 (0.55–1.54)	0.64 (0.28–1.50)

^a^In current pregnancy. Married-NLT – Married but not living together, Married-LT – married and living together, GA – gestational age, ANC – antenatal care, BMI – body mass index. OR Interpretation – ‘predictor name’ was associated with ‘OR’ increase (> 1) or decrease (< 1) in likelihood of belonging to ‘weight trajectory name’ compared to consistent medium trajectory class e.g. pre-pregnancy BMI-adjusted age category 25–29 years was associated with 3.87 increase in the likelihood of having medium–high weight trajectory compared to consistent medium trajectory class

To account for the contribution of estimated pre-pregnancy BMI to the observed associations, we included it in multivariable models (Table 2). In estimated pre-pregnancy BMI-adjusted analyses, continous Hb levels (aOR 1.58, 95% CI 1.10–2.25) remained positively associated with *consistent high* weight trajectory. Compared to normal Hb, severe anaemia (OR 1.14^–7^, 95% CI 2.05^–8^-6.32^–7^) was positively associated and living with HIV (aOR 0.45, 95% CI 0.21–0.95) remained inversely associated with *consistent high* weight trajectory compared to not living with HIV. Although the trend for continous Hb and categorical severe anaemia are in the same direction, this is not the case for moderate anaemia which shows a negative relationship with *consistent high* class membership.

### Maternal weight trajectory class and infant growth

At delivery, the proportion of infants born LBW was 10% and 4% had high birth weight (Table S2). Overall, 11% of infants were born SGA and 10% were born LGA. The proportion of spontaneous (11%) and medically indicated (10%) preterm births were similar. Overall, a high proportion (96%) of infants were breastfed from birth. However, HIV-exposed uninfected (HEU) infants were less likely to be breastfed for 6 months or longer compared to HIV-unexposed uninfected (HUU) infants (36% vs 64%, *p*-value 0.01). In descriptive analysis of z-scores at 12 months, overall mean WAZ was 0.52 (SD = 1.36), LAZ was -0.61 (SD = 1.28) and WLZ was 1.09 (SD = 1.54). HEU infants had significantly lower mean WAZ (0.39 SD = 1.35 vs 0.64 SD = 1.36, p-value 0.02) and LAZ (-0.77 SD = 1.23 vs -0.46 SD = 1.31, *p*-value 0.01), but there were no apparent differences in mean WLZ (1.01 SD = 1.51 vs 1.15 SD = 1.58, p-value 0.21) compared to HUU infants. Infants born from women with *consistent high* weight trajectory were more likely to be LGA (20% vs 7%, *p*-value 0.03) compared to those born from mothers with *consistent medium* weight trajectory (Table S3).

Variables included in adjusted models were maternal age, pre-pregnancy BMI, SES, parity, alcohol use, infant gender, gestation at birth and breastfeeding duration. In adjusted analysis, overall, only associations with infant WAZ remained statistically significant (Table 3). Compared to *consistent medium* weight trajectory, infants born from mothers with *consistent low* weight trajectory had decreased (mean difference -0.41, 95% CI -0.71;-0.12) WAZ at 12 months of age; and those born from mothers with *consistent high* weight trajectory had increased (mean difference 0.59, 95% CI 0.09;1.09) WAZ at 12 months of age. Associations between *consistent low* weight trajectory and decreased infant WAZ remained significant in both WLHIV (mean difference -0.30, 95% CI -0.70;-0.10) and those without HIV (mean difference -0.53, 95% CI -0.99;-0.08).

**Table 3:** Linear regression for the association between maternal weight trajectory classes (reference = *consistent medium*) and infant anthropometry at 12 months, overall and stratified by maternal HIV status

					**HIV status**					
		**Overall**				**Without HIV**			**With HIV**	
			**Unadjusted**	**Adjusted**		**Unadjusted**	**Adjusted**		**Unadjusted**	**Adjusted**
**Maternal weight class**	**Infant growth at 12 months**	**Total N**	**Mean diff. (95% CI)**	**Mean diff. (95% CI)**	**Total N**	**Mean diff. (95% CI)**	**Mean diff. (95% CI)**	**TotalN**	**Mean diff. (95% CI)**	**Mean diff. (95% CI)**
***CONSISTENT LOW***	Weight for age Z-score (WAZ)	613	**-0.46 (-0.72;-0.21)**	**-0.41 (-0.71;-0.12)**	315	**-0.51 (-0.87;-0.16)**	**-0.53 (-0.99;-0.08)**	298	**-0.42 (-0.78;-0.06)**	**-0.30 (-0.70;-0.10)**
	Length for age Z-score (LAZ)	613	**-0.32 (-0.59;-0.04)**	**-0.40 (-0.72;-0.08)**	315	-0.30 (-0.71;0.10)	-0.48 (-0.98;0.01)	298	-0.34 (-0.71;0.03)	-0.30 (-0.70;0.11)
	Weight for length Z-score (WLZ)	613	**-0.38 (-0.68;-0.09)**	-0.28 (-0.63;0.08)	315	**-0.48 (-0.91;-0.05)**	-0.40 (-0.94;0.14)	298	-0.29 (-0.70;0.12)	-0.18 (-0.64;0.27)
***MEDIUM HIGH***	Weight for age Z-score (WAZ)	613	**0.36 (0.09;0.63)**	0.21 (-0.11;0.54)	315	0.29 (-0.08;0.66)	0.19 (-0.25;0.62)	298	0.39 (-0.01;0.79)	0.21 (-0.27;0.69)
	Length for age Z-score (LAZ)	613	0.14 (-0.11;-0.39)	0.22 (-0.08;0.52)	315	0.01 (-0.35;0.37)	0.12 (-0.29;0.53)	298	0.25 (-0.10;0.60)	0.28 (-0.17;0.74)
	Weight for length Z-score (WLZ)	613	**0.39 (0.10;0.69)**	0.15 (-0.20;0.51)	315	0.39 (-0.01;0.78)	0.17 (-0.31;0.66)	298	0.37 (-0.06;0.81)	0.12 (-0.41;0.66)
***CONSISTENT HIGH***	Weight for age Z-score (WAZ)	613	**0.67 (0.28;1.07)**	**0.59 (0.09;1.09)**	315	**0.62 (0.06;1.18)**	0.58 (-0.12;1.28)	298	**0.68 (0.14;1.22)**	0.49 (-0.21;1.19)
	Length for age Z-score (LAZ)	613	0.05 (-0.27;0.37)	0.38 (-0.11;0.88)	315	0.10 (-0.36;0.56)	0.47 (-0.20;1.13)	298	-0.12 (-0.54;0.31)	0.12 (-0.60;0.84)
	Weight for length Z-score (WLZ)	613	**0.89 (0.41;1.36)**	0.57 (-0.04;1.17)	315	**0.79 (0.10;1.48)**	0.48 (-0.36;1.32)	298	**0.98 (0.37;1.60)**	0.62 (-0.21;1.45)

The beta coefficient (**β**) represents mean difference. Interpretation—‘weight trajectory name’ was associated with ‘ **Mean diff.**’ unit increase ( +) or decrease (-) in outcome e.g. consistent high weight trajectory was associated with 0.59 unit increase in infant WAZ at 12 months of age. Models were adjusted for maternal (estimated pre-pregnancy BMI, parity, SES and alcohol use) and infant (sex, delivery GA and feeding duration) factors

## Discussion

In this cohort of women in an urban setting in South Africa, we identified four distinct patterns of weight trajectories from pregnancy through to 12 months postpartum, ranging from consistently low to consistently high. Infants born from women with *consistent low* weight trajectory had decreased WAZ; while those born from women with *consistent high* weight trajectory had increased WAZ at 12 months of age. These findings demonstrate the relationship between longitudinal maternal weight and infant growth, and could help inform the design of adaptive weight management interventions.

Pre-pregnancy BMI is traditionally used to categorise individuals in order to guide weight management interventions. While this is useful, in the context of pregnancy increased GWG and PPW are also of public health concern in addition to pre-pregnancy BMI. This study used a weight trajectory approach to detect women with ‘abnormal’ weight progression over time. We found that women with normal estimated pre-pregnancy BMI were assigned to *consistent low* and some to *consistent medium* trajectories due to different weight progression trends over time. In agreement, using a trajectory approach, Bogaerts et al. [52] found that some lean women experienced excessive GWG and retained the highest amount of weight after delivery compared to those who started off with obese BMI. This trajectory approach allowed these lean women with excessive GWG, who would otherwise be excluded from targeted weight management interventions, to be identified as high risk for adverse health outcomes. Considering the high fertility and multiple pregnancies with short birth intervals in sub-Saharan Africa [53], identification of high risk women using a comprehensive trajectory method might be useful for planning inclusive weight management interventions.

Maternal exposures during pregnancy influence fetal development, thereby setting life course health outcomes for offspring. Despite the evidence showing the association between pre-pregnancy BMI or GWG and adverse infant outcomes [19, 54] there are limited data on the associations with maternal weight patterns over time. Catov et al., [55] highlighted the usefulness of studying weight as a trajectory over time by showing that the influence of GWG on infant weight is dependent on the timing in which the weight change occurred. The authors found that women who experienced excessive weight gain before 20 weeks of pregnancy were more likely to have LGA infants, with a recommendation that weight gain pattern throughout the course of pregnancy might be a useful marker of abnormal fetal growth [55]. In this cohort we found that *consistent low* trajectory was associated with decreased, while *consistent high* trajectory was associated with increased infant WAZ at 12 months, regardless of maternal HIV status. Notably, the majority (72%) of women with *consistent low* weight trajectory had normal pre-pregnancy BMI. Using cross-sectional BMI as an exposure, these women would not be identified as being at risk of having infants with non-optimal growth patterns. Poor infant growth during the first 1000 days lays the foundation for risk of reduced human capital and economic productivity due to poor brain development [56]; as well as risk of chronic metabolic diseases such as obesity and diabetes over life-course [57]. Therefore, inclusive detection of women at risk of adverse weight trajectories is necessary for inclusive interventions directed at reducing adverse infant outcomes.

Indeed, identification of risks factors contributing to ‘abnormal’ weight patterns was carried out in this study. We found that women with *medium high* and *consistent high* weight trajectories were more likely to be older and to have increased BMI, blood pressure, haemoglobin levels and parity. These results highlight the type of women in which weight management programs should be targeted to before and during pregnancy in low-resource settings to minimise adverse infant growth outcomes. Fortunately, all these factors are examined routinely at the first ANC visit in South African public sector antenatal clinics. This means that the applicability of these findings in this setting would be feasible. For example, this could involve communication of risk to patient by the healthcare practitioners to raise early awareness, followed by close monitoring of weight gain throughout pregnancy. Further, counselling regarding the importance of healthy postpartum weight retention would be essential to minimise the long-term risk of adverse maternal health outcomes.

Caution should be exercised when interpreting these study findings due to certain limitations. Women were enrolled at first ANC which was mainly in the second trimester of pregnancy and hence pre-pregnancy weight was not known. Although pre-pregnancy weight was estimated, there is still potential for misclassification bias in the variables that include this corrected weight such as pre-pregnancy BMI and PPW variables. The GWG charts used to apply a correction factor on measured first ANC weight to estimate pre-pregnancy BMI were developed using international standards which do not include WLHIV and no data exist for this group. However, considering the similar weight trajectories observed among both women living with and those without HIV, substantial differences in the GWG trends are unlikely. Other important weight trajectory predictors such as mental health and physical activity were not measured. Not all infants could be included in the study, this missing data may have led to selection bias. Despite the intuitive nature of weight trajectory analytical approach compared to cross-sectional BMI, some subtypes of weight trajectory classes may not have been identified due to missing weight assessments at some visits and due to a limited sample size, and large-scale studies are needed. However, longitudinal follow-up of mother-infant pairs throughout pregnancy to 12 months postpartum is a unique strength.

In conclusion, this study is among the first to identify distinct maternal weight trajectories in African women. *Consistent low* weight trajectory was associated with decreased, while *consistent high* was associated with increased infant WAZ at 12 months of age. A longitudinal weight trajectory approach might inform comprehensive efforts targeted at improving healthy maternal weight and infant outcomes.

### Supplementary Information

**Additional file 1.**

**Additional file 2.**

**Additional file 3.**

**Additional file 4.**

**Additional file 5.**

## Data Availability

All data generated or analysed during this study are included in this published article (and its supplementary information files). In addition, the datasets used and/or analysed during the current study are available from the corresponding author on reasonable request.

## Abbreviations

BMI: Body mass index
GWG: Gestational weight gain
PPW: Postpartum weight
WAZ: Weight-for-age
LAZ: Length-for-age
WLZ: Weight-for-length
OR: Odds ratio
WLHIV: Women living with HIV
ART: Antiretroviral therapy
NCDs: Non-communicable diseases
CVDs: Cardiovascular diseases
LGA: Large for gestational age
SGA: Small for gestational age
ANC: Antenatal care
SES: Socioeconomic status
WHO: World Health Organisation
IOM: Institute of Medicine
BP: Blood pressure
Hb: Haemoglobin
AUDIT: Alcohol Use Disorders Identification Test
GA: Gestational age
PTD: Preterm delivery
BIC: Bayesian Information Criterion
