# Supplementary material for: Maternal weight trajectories and associations with infant growth in South African women
Source: BMC Public Health. 2023 Oct 20;23:2055. doi: 10.1186/s12889-023-16963-3 (PMC10588171; doi:10.1186/s12889-023-16963-3)
Supplement: Supplementary file 1 — Additional file 1. [file 12889_2023_16963_MOESM1_ESM.pdf]

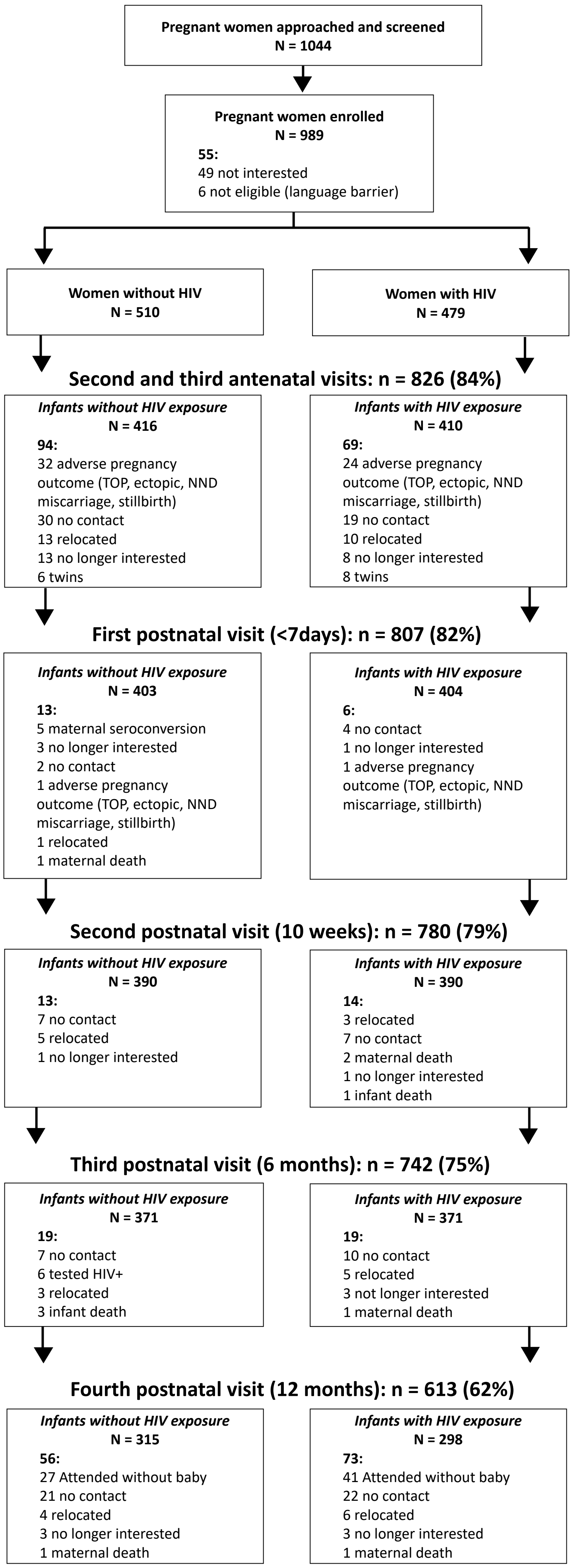

Figure S1. Flow diagram showing participant retention and selection of infants included for growth analysis
