# Supplementary material for: Maternal weight trajectories and associations with infant growth in South African women
Source: BMC Public Health. 2023 Oct 20;23:2055. doi: 10.1186/s12889-023-16963-3 (PMC10588171; doi:10.1186/s12889-023-16963-3)
Supplement: Supplementary file 2 — Additional file 2. [file 12889_2023_16963_MOESM2_ESM.pdf]

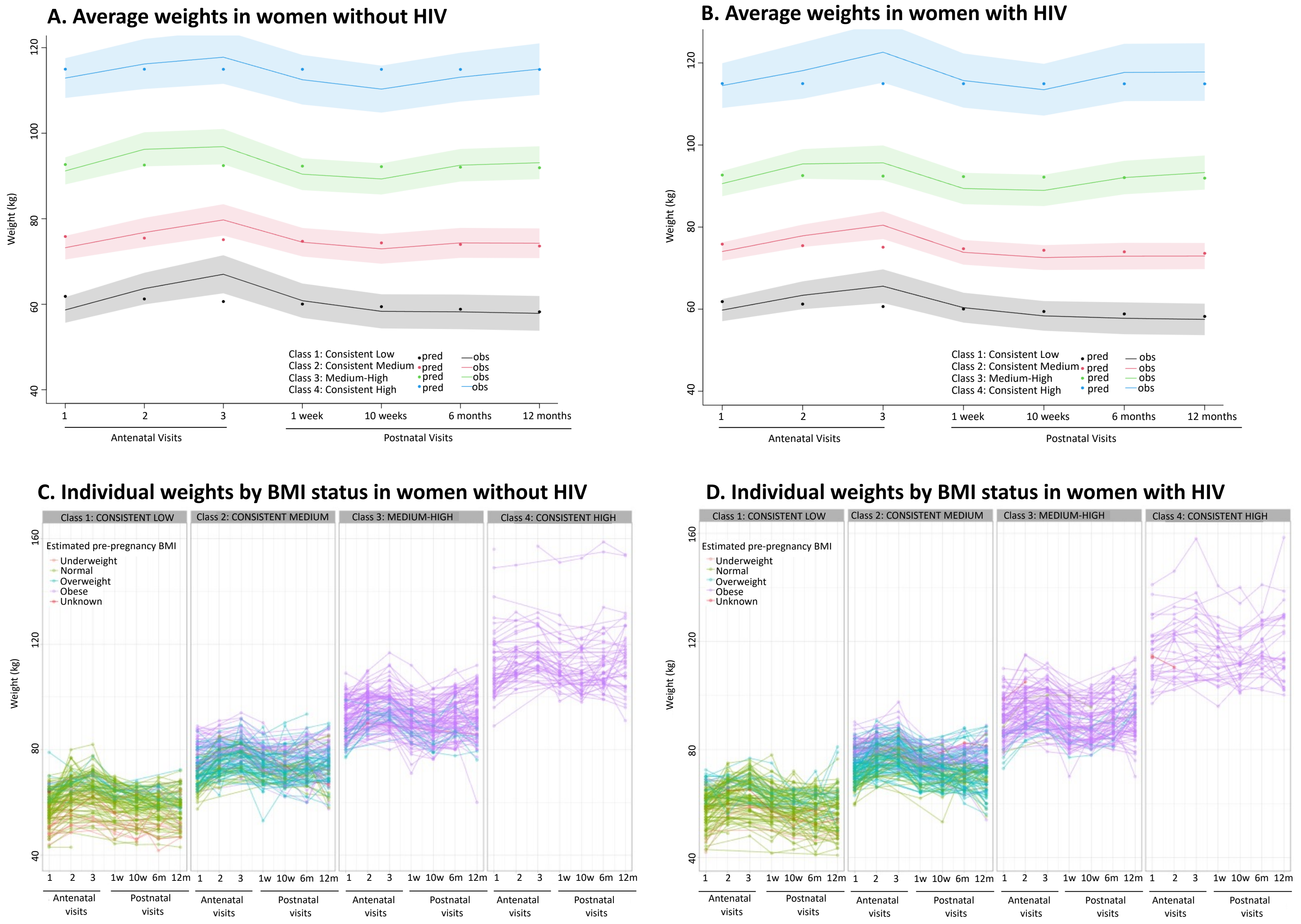

Figure S2. Four distinct maternal weight trajectory classes, A. average weights among women without HIV, B. average weights among women with HIV, C. individual weights by estimated pre-pregnancy BMI among women without HIV and D. individual weights by estimated pre-pregnancy BMI among women with HIV.
