# Supplementary material for: Maternal weight trajectories and associations with infant growth in South African women
Source: BMC Public Health. 2023 Oct 20;23:2055. doi: 10.1186/s12889-023-16963-3 (PMC10588171; doi:10.1186/s12889-023-16963-3)
Supplement: Supplementary file 3 — Additional file 3. [file 12889_2023_16963_MOESM3_ESM.docx]

| Table S1. Characteristics of women included in the analysis, overall and stratified by maternal weight trajectory class |
| --- |

|  |  | Weight trajectory class | | | |  |
| --- | --- | --- | --- | --- | --- | --- |
|  | Overall  N=989 (100%)  N (%) | *Consistent low*  N=283 (29%)  N (%) | *Consistent medium*  N=366 (37%)  N (%) | *Medium high*  N=238 (24%)  N (%) | *Consistent high*  N=102 (10%)  N (%) | p-value |
| Age (years)  <24  25-29  30-34  ≥35  Median (IQR) | 240 (24)  292 (30)  254 (26)  203 (21)  29 (25-34) | 94 (32)  90 (32)  69 (24)  30 (11)  27 (23-32) | 93 (25)  101 (28)  83 (23)  89 (24)  29 (24-34) | 38 (16)  80 (34)  66 (28)  54 (23)  30 (26-34) | 15 (15)  21 (21)  36 (35)  30 (29)  32 (27-35) | **<0.01** |
| Blood pressure (mmHg)  Normal  Elevated  Stage 1 hypertension  Stage 2 hypertension  Median SBP (IQR)  Median DBP (IQR) | 620 (63)  160 (16)  108 (11)  101 (10)  113 (105-122)  67 (61-73) | 209 (74)  27 (10)  20 (7)  27 (10)  109 (102-116)  65 (60-70) | 238 (65)  59 (16)  29 (8)  40 (11)  113 (106-121)  66 (60-72) | 128 (54)  51 (21)  41 (17)  18 (8)  117 (110-126)  70 (63-75) | 45 (44)  23 (23)  18 (18)  16 (16)  121 (111-130)  70 (64-78) | **<0.01** |
| Haemoglobin (g/dL)  Normal (≥11.0)  Mild anaemia (10-10.9)  Moderate anaemia (7-9.9)  Severe anaemia (<7)  Missing  Median (IQR) | 394 (40)  150 (15)  114 (12)  6 (1)  325 (33)  11.3 (10.4-12.1) | 86 (30)  46 (16)  41 (14)  4 (1)  106 (37)  10.9 (9.9-11.8) | 139 (38)  58 (16)  48 (13)  1 (1)  120 (33)  11.2 (10.3-12.0) | 116 (49)  35 (15)  17 (7)  1 (1)  69 (29)  11.5 (10.6-12.2) | 53 (52)  11 (11)  8 (8)  0  30 (29)  11.7 (10.9-12.6) | **0.01** |
| Education  Primary  High school  Tertiary | 37 (4)  929 (94)  23 (2) | 9 (3)  267 (94)  7 (2) | 16 (4)  343 (94)  7 (2) | 6 (3)  225 (95)  7 (3) | 6 (6)  94 (92)  2 (2) | 0.73 |
| Socio-economic status  Lower  Middle  Higher  Missing | 325 (33)  274 (28)  388 (39)  2 (0.2) | 98 (35)  75 (27)  110 (39)  0 (0) | 125 (34)  108 (30)  131 (36)  2 (1) | 64 (27)  66 (28)  108 (43)  0 (0) | 38 (37)  25 (25)  39 (38)  0 (0) | 0.25 |
| Relationship status  No relationship  Not Cohabiting/married-NLT  Cohabiting/married-LT  Missing | 46 (5)  509 (51)  428 (43)  6 (1) | 15 (5)  168 (59)  97 (34)  3 (1) | 20 (5)  185 (51)  160 (44)  1 (1) | 9 (4)  108 (45)  119 (50)  2 (1) | 2 (2)  48 (47)  52 (51)  0 (0) | **0.02** |
| *Alcohol use  No  Yes  Missing | 898 (91)  89 (9)  2 (0.2) | 250 (88)  32 (11)  1 (0.4) | 334 (91)  32 (9)  0 (0) | 219 (92)  18 (8)  1 (0.4) | 95 (93)  7 (7)  0 (0) | 0.56 |
| GA at first ANC (weeks)  1^st^ trimester (≤13)  2^nd^ trimester (14-28)  3^rd^ trimester (>28)  Missing  Median (IQR) | 229 (23)  609 (62)  123 (12)  28 (3)  20 (14-25) | 64 (23)  176 (62)  31 (11)  12 (4)  20 (14-25) | 74 (20)  236 (64)  46 (13)  10 (3)  20 (15-24) | 60 (25)  147 (62)  27 (11)  4 (2)  19 (13-25) | 31 (30)  50 (49)  19 (19)  2 (2)  20 (13-26) | 0.11 |
| Parity  Primiparity  Multiparity  Median (IQR) | 251 (25)  738 (75)  1 (0-2) | 103 (36)  180 (64)  1 (0-2) | 86 (24)  280 (77)  1 (1-2) | 50 (21)  188 (79)  1 (1-2) | 12 (12)  90 (88)  2 (1-2) | **<0.01** |
| HIV status  Without HIV  With HIV | 510 (52)  479 (48) | 148 (52)  135 (48) | 166 (45)  200 (55) | 131 (55)  107 (45) | 65 (64)  37 (36) | **0.01** |
| ART initiation timing  Pre-pregnancy  During pregnancy | 291 (61)  188 (39) | 74 (276  61 (21) | 119 (33)  81 (22) | 72 (30)  35 (15) | 26 (25)  11 (11) | **0.01** |
| CD4 count (cells/µL)  Median (IQR)  Missing | 458 (311-604)  56 (12) | 379 (259-511)  17 (13) | 494 (346-616)  26 (13) | 504 (389-668)  10 (9) | 498 (299-671)  3 (8) | **<0.01** |
| Food intake in past 4-7 days  Starch  Protein  Dairy  Fruits  Vegetables  Legumes  Oils  Missing | 523 (53)  285 (29)  177 (18)  131 (13)  289 (29)  79 (8)  386 (39)  3 (0.3) | 138 (49)  84 (30)  45 (16)  34 (12)  71 (25)  21 (7)  100 (35)  2 (1) | 203 (55)  106 (29)  71 (19)  55 (15)  113 (31)  33 (9)  150 (41)  0 | 131 (55)  67 (28)  38 (16)  30 (13)  68 (29)  16 (7)  96 (40)  1 (0.4) | 51 (50)  28 (27)  23 (23)  12 (12)  37 (36)  9 (9)  40 (39)  0 | 0.11  0.59  0.13  0.84  0.31  0.13  0.76 |
| Estimated pre-pregnancy BMI (kg/m^2^)  Underweight (<18.5)  Normal (18.5-24.9)  Overweight (25-29.9)  Obese (≥30)  Missing  Median (IQR) | 21 (2)  273 (28)  244 (25)  428 (43)  23 (2)  29 (24-34) | 21 (7)  203 (72)  43 (15)  6 (2)  10 (4)  22 (20-24) | 0 (0)  68 (19)  180 (49)  110 (30)  8 (2)  28 (26-31) | 0 (0)  2 (1)  21 (9)  213 (90)  2 (1)  35 (32-38) | 0 (0)  0 (0)  0 (0)  99 (97)  3 (3)  42 (39-46) | **<0.01** |
| Gestational weight gain rate (kg/week)  Slow  Normal  Fast  Missing  Median (IQR) | 264 (27)  121 (12)  361 (37)  243 (25)  0.36 (0.14-0.54) | 90 (32)  48 (17)  66 (23)  79 (28)  0.37 (0.16-0.55) | 97 (27)  47 (13)  140 (38)  82 (22)  0.35 (0.13-0.53) | 53 (22)  22 (9)  110 (46)  53 (22)  0.36 (0.15-0.54) | 24 (24)  4 (4)  45 (44)  29 (28)  0.38 (0.08-0.55) | **<0.01** |
| Postpartum weight at 12 months (kg)  Weight loss (<0kg)  Stable weight retention (0-4.9kg)  High weight retention (≥5kg)  Missing  Median (IQR) | 209 (21)  178 (18)  300 (30)  302 (31)  3.87 (-1.10, 9.04) | 55 (19)  58 (20)  68 (24)  102 (36)  2.98 (-0.87, 7.75) | 83 (23)  57 (16)  113 (31)  113 (31)  3.95 (-1.94, 9.21) | 48 (20)  40 (17)  92 (37)  58 (24)  5.11 (-0.48, 9.43) | 23 (23)  23 (23)  27 (26)  29 (28)  1.73 (-1.35, 8.91) | **0.02** |

| * In current pregnancy. Married-NLT – Married but not living together, Married-LT – married and living together, GA – gestational age, ANC – antenatal care, BMI – body mass index. |
| --- |
