# Supplementary material for: Maternal weight trajectories and associations with infant growth in South African women
Source: BMC Public Health. 2023 Oct 20;23:2055. doi: 10.1186/s12889-023-16963-3 (PMC10588171; doi:10.1186/s12889-023-16963-3)
Supplement: Supplementary file 4 — Additional file 4. [file 12889_2023_16963_MOESM4_ESM.docx]

Table S2. Characteristics of infants included in the analysis, overall and stratified by *in utero* HIV exposure status

|  |  | HIV exposure status | |  |
| --- | --- | --- | --- | --- |
|  | Overall  N (%) = 613 | HUU  N (%) = 315 (51%) | HEU  N (%) = 298 (49%) | p-value |
| Gender  Male  Female | 304 (50)  309 (50) | 155 (49)  160 (51) | 154 (52)  144 (48) | 0.54 |
| Birthweight (g)  Low (<2500)  Normal (2500-4000)  High (>4000)  Median (IQR) | 62 (10)  525 (86)  26 (4)  3180 (2880-3460) | 27 (9)  271 (86)  17 (5)  3220 (2940-3520) | 35 (12)  254 (85)  9 (3)  3130 (2800-3420) | 0.17 |
| Size for GA (percentile)  Small (<10^th^)  Appropriate (10-90^th^)  Large (>90^th^) | 67 (11)  484 (79)  62 (10) | 28 (9)  248 (79)  39 (12) | 39 (13)  236 (79)  23 (8) | 0.06 |
| Gestation at delivery (weeks)  Spontaneous preterm (<37)  Medically-indicated preterm (<37)  Term delivery (≥37)  Missing | 66 (11)  61 (10)  464 (76)  22 (4) | 31 (10)  30 (10)  247 (78)  7 (2) | 35 (12)  31 (10)  217 (73)  15 (5) | 0.20 |
| Breastfeeding duration (months)  Never  Ever  <6 months  ≥6 months  Missing  Median (IQR) | 14 (2)  589 (96)  294 (48)  309 (50)  10 (2)  6 (2-12) | 3 (1)  307 (97)  109 (35)  201 (64)  5 (2)  9 (3-12) | 11 (4)  282 (95)  185 (62)  108 (36)  5 (2)  2 (1-6) | 0.08  **<0.01** |
| Z-scores at 12 months, mean (SD)  Weight-for-age (WAZ)  Length-for-age (LAZ)  Weight-for-length (WLZ) | 0.52 (1.36)  -0.61 (1.28)  1.09 (1.54) | 0.64 (1.37)  -0.46 (1.31)  1.15 (1.58) | 0.39 (1.35)  -0.77 (1.23)  1.01 (1.51) | **0.02**  **0.01**  0.21 |

HUU - HIV-unexposed uninfected, HEU - HIV-exposed uninfected, GA - gestational age
