# Supplementary material for: Maternal weight trajectories and associations with infant growth in South African women
Source: BMC Public Health. 2023 Oct 20;23:2055. doi: 10.1186/s12889-023-16963-3 (PMC10588171; doi:10.1186/s12889-023-16963-3)
Supplement: Supplementary file 5 — Additional file 5. [file 12889_2023_16963_MOESM5_ESM.docx]

| Table S3. Characteristics of infants included in the analysis, overall and stratified by maternal weight trajectory class |
| --- |

|  |  | Maternal weight trajectory class | | | |  |
| --- | --- | --- | --- | --- | --- | --- |
|  | Overall  N=613 (100%)  N (%) | *Consistent low*  N=154 (25%)  N (%) | *Consistent medium*  N=225 (37%)  N (%) | *Medium-high*  N=164 (27%)  N (%) | *Consistent high*  N=70 (11%)  N (%) | p-value |
| Gender  Male  Female | 309 (50)  304 (50) | 75 (49)  95 (51) | 110 (49)  115 (51) | 85 (52)  79 (48) | 39 (56)  31 (44) | 0.73 |
| Birthweight (g)  Low (<2500)  Normal (2500-4000)  High (>4000)  Median (IQR) | 62 (10)  525 (86)  26 (4)  3180 (2880-3460) | 24 (16)  128 (83)  2 (1)  3025 (2710-3260) | 18 (8)  198 (88)  9 (4)  3170 (2910-3440) | 11 (7)  143 (87)  10 (6)  3315 (2970-3550) | 9 (13)  56 (80)  5 (7)  3345 (2960-3640) | **0.03** |
| Size for GA (percentile)  Small (<10^th^)  Appropriate (10-90^th^)  Large (>90^th^) | 67 (11)  484 (79)  62 (10) | 23 (15)  121 (79)  10 (7) | 24 (11)  185 (82)  16 (7) | 14 (9)  128 (78)  22 (13) | 6 (9)  50 (71)  14 (20) | **0.01** |
| Gestation at delivery (weeks)  Spontaneous preterm (<37)  Medically-indicated preterm (<37)  Term delivery (≥37)  Missing | 66 (11)  61 (10)  464 (76)  22 (4) | 24 (16)  14 (9)  110 (71)  6 (4) | 25 (11)  22 (10)  167 (74)  11 (5) | 13 (8)  14 (9)  133 (81)  4 (2) | 4 (6)  11 (16)  54 (77)  1 (1) | 0.18 |
| Breastfeeding duration (months)  Never  Ever  <6 months  ≥6 months  Missing  Median (IQR) | 14 (2)  589 (96)  294 (48)  309 (50)  10 (2)  6 (2-12) | 3 (2)  149 (97)  79 (51)  73 (47)  2 (1)  4 (2-12) | 5 (2)  216 (96)  116 (52)  105 (47)  4 (2)  5 (2-12) | 4 (2)  157 (96)  66 (40)  95 (58)  3 (2)  6 (2-12) | 2 (3)  67 (96)  33 (47)  36 (51)  1 (1)  6 (2-12) | 1.00  0.43 |
| Z-scores at 12 months, mean (SD)  Weight for age  Length for age  Weight for length | 0.52 (1.36)  -0.61 (1.28)  1.09 (1.54) | 0.01 (1.20)  -0.89 (1.42)  0.59 (1.42) | 0.46 (1.27)  -0.57 (1.20)  0.98 (1.45) | 0.82 (1.38)  -0.43 (1.26)  1.37 (1.44) | 1.14 (1.50)  -0.53 (1.19)  1.86 (1.86) | **0.01**  0.05  **0.01** |

| GA – gestational age |
| --- |
